# Early spinogenesis in postnatally-born hippocampal granule cells

**DOI:** 10.3389/fnana.2026.1899044

**Published:** 2026-09-30

**Authors:** Lukas Frey, Sophia Kirscht, Judith Ebert, Tijana Hanauske, Thomas Deller, Stephan W. Schwarzacher, Tassilo Jungenitz

**Affiliations:** Institute of Clinical Neuroanatomy, Neuroscience Center, Goethe-University Frankfurt, Frankfurt Am Main, Germany

**Keywords:** dendritic development, dentate gyrus, neurogenesis, postnatally-born granule cells, synaptogenesis

## Abstract

**Introduction:**

The development of early postnatally-born hippocampal granule cells (pbGCs) can be studied in organotypic entorhino-hippocampal slice cultures (OTCs) in which local dentate gyrus (DG) and entorhinal projections are established within the first 10 days *in vitro*. Thus, pbGCs develop within an existing neural network, comparable to the adult situation *in vivo.*

**Methods:**

In this study, we analyzed the development of small dendritic protrusions (SDPs) on complete reconstructions of dendritic trees from retrovirally (RV) labeled pbGC born at postnatal day 4–5 during the phase of early spinogenesis in OTCs.

**Results:**

We found SDPs with head (spines) and without head (other protrusions) as early as 12 days post RV injection (dpi) in all parts of the growing dendritic tree of pbGCs. At 14 – 20 dpi, while dendritic reorganization was still ongoing, SDP density as well as spine tip size increased moderately. At 16 dpi, spines with larger, bulbous heads appeared. Interestingly, the percentage of spines stayed constant at 60% of all SDPs over time. While spines were evenly distributed in all parts of the dendritic tree with no preference for any layer, other SDPs were more abundant at proximal parts of the dendritic tree within the granule cell layer (GCL).

**Discussion:**

Our findings support the idea that SDPs appear early in dendritic development and are associated with dendritic growth rather than the establishment of specific synaptic connectivity. The uniform distribution of spines across the dendritic tree during the phase of dendritic maturation and reorganization suggests that early spinogenesis may be guided in part by intrinsic cellular programs, while not excluding a contribution of layer-specific cues.

## 1 Introduction

Hippocampal granule cells (GCs, see Table 1 for a list of abbreviations) are generated from late embryonic development on, exhibit a peak of proliferation in the early postnatal period and continue to be generated throughout life within the subgranular zone (SGZ) of the dentate gyrus (DG; Altman and Bayer, 1990a,1990b; Schlessinger et al., 1975). New GCs generated from neural stem cells in the SGZ extend their dendrites into the molecular layer (ML) where they contact axons from local neurons and incoming external afferents to form synaptic connections and become integrated in the existing neural network. Extensive work from the past decades have provided evidence for a substantial role of functionally integrated adult-born granule cells (abGCs) in hippocampus-dependent functions related to learning and memory, mood regulation and various human pathophysiological conditions (reviewed in Denoth-Lippuner and Jessberger, 2021). However, the extent to which synapse formation and integration of new GCs depend on specific afferent input from the existing network is still not fully understood. The laminar organization of the entorhinal and local DG projections is preserved in postnatal entorhino-hippocampal slice cultures (OTCs; Hildebrandt-Einfeldt et al., 2021; Kluge et al., 1998; Li et al., 1994) and additionally, postnatally-born GCs (pbGCs) of the SGZ can be visualized by retroviral labeling (Radic et al., 2017). Therefore, OTCs offer the distinct opportunity to study the dendritic maturation of pbGCs as well as their integration in an already established functional network (Kamada et al., 2004; Namba et al., 2011; Radic et al., 2017; Raineteau et al., 2006, 2004). Although pbGCs are generated in the early postnatal period, they resemble abGCs in many characteristics, albeit their maturation is apparently protracted in adulthood (Cole et al., 2020; Jungenitz et al., 2018; Ohkawa et al., 2012; Radic et al., 2017; Zhao et al., 2006). Comparable to abGCs, pbGCs undergo several dendritic maturation steps before they start to gradually become integrated into the hippocampal network within 5 weeks of cellular age (Radic et al., 2017). Interestingly, small dendritic protrusions (SDPs), as structural equivalents of maturation and synaptic connectivity, begin to emerge at the end of the second week of cell development during the phase of highly dynamic dendritic growth and become more numerous over time (Radic et al., 2017).

**TABLE 1:** List of abbreviations.

Abbreviation	Definition	Abbreviation	Definition
abGCs	Adult-born granule cells	MML	Middle molecular layer
DG	Dentate gyrus	OML	Outer molecular layer
dpi	Days post injection	OTCs	Organotypic entorhino-hippocampal slice cultures
EC	Entorhinal cortex	pbGCs	Postnatally-born granule cells
GCL	Granule cell layer	RV	Retrovirus
GCs	Granule cells	SDPs	Small dendritic protrusions
IML	Inner molecular layer	SGZ	Subgranular zone
ML	Molecular layer	TDL	Total dendritic length

Here, we investigated at which time point of cellular development the first SDPs, including spines, can be detected on pbGC dendrites, and whether early spinogenesis exhibits any local preferences to certain laminae of the DG. We found an early onset of SDPs and a rather even distribution of spines on all parts of the dendritic tree. While we cannot exclude the influence of layer-specific extrinsic cues, our findings suggest that dendritic spine development is established with a strong contribution from intrinsic developmental programs during structural maturation.

## 2 Materials and methods

### 2.1 Animals

In the present study we used OTCs derived from neonatal P4/P5 Wistar rats of both sexes as an *in vitro* model to study the structural development of pbGCs. Sex determination was not performed for this study. Parent animals were acquired by Jackson Laboratories and paired for offspring at the “Zentrale Forschungseinrichtung” at the Goethe University Hospital in Frankfurt am Main. Animals were housed on a 12-h day/night cycle with food and water available at all times. Experimental procedures involving animals were performed in accordance with the German animal welfare law on the use of laboratory animals and had been declared to the Animal Welfare Officer of the University (Ref. No TSB 2024-11-01). The methods used to kill animals were consistent with the Directive 2010/63/EU. Every effort was made to minimize distress and pain for animals.

### 2.2 Preparation of entorhino-hippocampal slice cultures (OTCs)

For the preparation of OTCs, we followed a protocol established by Del Turco and Deller, 2007 which is an adaptation of the Stoppini-preparation method (Stoppini et al., 1991). In brief, neonatal P4/5 rat pups were killed by rapid decapitation. The brain was removed and glued onto a microtome plate using Histoacryl^®^ tissue glue (B. Braun SE, Melsungen, Germany). The plate was quickly moved into the sectioning chamber of the microtome (VT1200S, Leica, Wetzlar, Germany) which contained ice cold preparation medium consisting of 95% MEM, 0.65% glucose, 25 mM HEPES, Glutamax 2 mM (all from Gibco, Invitrogen, Waltham, MA, USA), streptomycin 0.1 mg/ml and penicillin 100 U/ml (both from Sigma-Aldrich, St. Louis, MO, USA). Two to three 300 μm thick horizontal brain sections in the middle of the septo-temporal axis were acquired and transferred to a 30 mm petri dish. Under a stereomicroscope (Stemi 2000-C, Zeiss, Oberkochen, Germany), the hippocampal formation including large parts of the entorhinal cortex (EC) were cut out of the brain slices using a scalpel. Two to four tissue preparations were then transferred onto a membrane (Millicell Standing Cell Culture Insert, Sigma-Aldrich, St. Louis, MO, USA) which was placed into a well of a 6 well-plate (Celstar 657160, Greiner Bio-One International GmbH, AT) containing 1 ml of warm pH-adjusted incubation medium (42% Minimum essential medium, 25% Basal Eagle Medium, 25% Heat-inactivated normal horse serum, 0.65% Glucose, 25 mM HEPES, 0.15% Sodium bicarbonate, 2 mM Glutamax (all from Gibco, Invitrogen, Waltham, MA, USA), 0.1 mg/ml Streptomycin and 100 U/ml Penicillin (both from Sigma-Aldrich, St. Louis, MO, USA). OTCs were cultivated in a humidified incubator (95% air, 5% CO_2_, 95% H_2_O-saturation, 35°C) and incubation medium was completely exchanged every 2–3 days.

### 2.3 Retroviral transductions

To label newborn neurons, we used a retroviral (RV) construct based on a Moloney murine leukemia virus encoding for a GFP tag under the CAG promoter which has been established by Van Praag et al., 2002. Retroviral injections were performed directly after preparation at DIV 0 into the subgranular zone of the DG, similarly to the method described by Radic et al., 2017 (Figure 1A). For viral application, an injection pipette with a diameter of 30 μm was pulled from glass capillaries (GC150-10, Harvard apparatus Ltd., Holliston, MA, USA) by a DMZ universal puller (Zeitz-Instruments Vertriebs GmbH, Martinsried, Germany). Approximately 0.2 μl of the virus lysate (with a viral titer of approximately 10^5^ colony-forming units) was pressure-injected (Transjector 5246, Eppendorf, Hamburg, Germany) into each OTC. For precise virus application, pipettes were placed under visual control using a micromanipulator (SM 325, Märzhäuser Wetzlar, Wetzlar, Germany) which was mounted on top of a modified Olympus BX50 light transmission microscope equipped with a camera system (ColorView II, controlled by AnalySIS 2677, both from Olympus, Tokyo, Japan).

**FIGURE 1:**
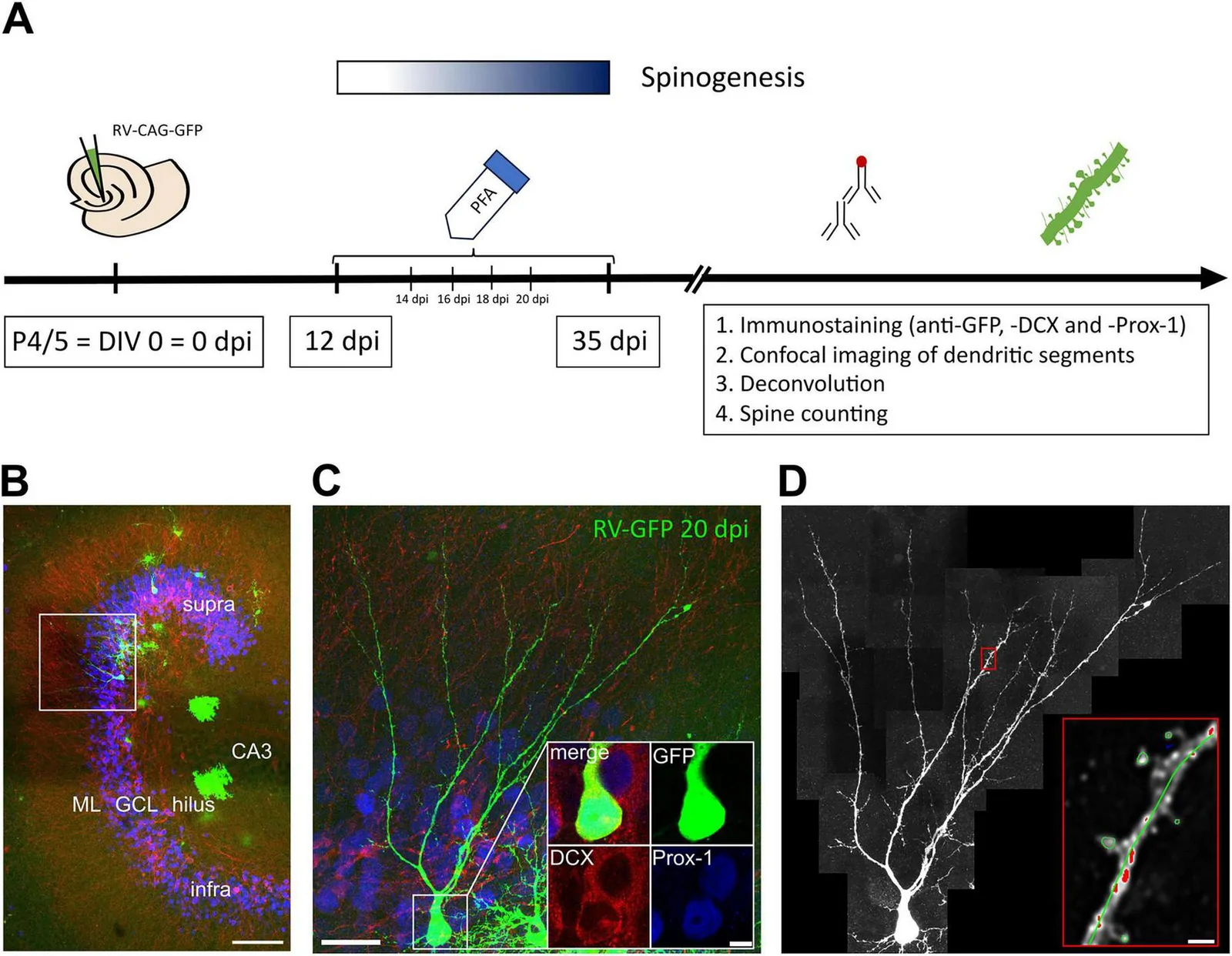
Structural analysis of retrovirally labeled postnatally-born granule cells (pbGCs) in organotypic entorhino-hippocampal slice cultures (OTCs). **(A)** Schematic diagram of experimental design: OTCs were prepared from rat brains at postnatal days 4/5. Retroviral injection (RV-CAG-GFP) into the subgranular zone (SGZ) of the dentate gyrus (DG) to label pbGCs was performed at the same day of preparation (DIV 0 = 0 dpi). Cultures were fixed at different time points ranging between 12 to 35 dpi. Immunohistochemistry was performed to enhance the green fluorescent protein (GFP), and to detect Doublecortin (DCX) and Prox-1, followed by confocal image acquisition, processing, and analysis. **(B)** Overview of the DG in an OTC after fixation and immunohistochemical staining for GFP (green), DCX (red), and Prox-1 (blue). **(C)** RV-GFP-labeled pbGC located in the SGZ of the DG at 20 dpi. Immunolabeling verified that this cell is positive for Prox-1 and DCX. **(D)** Multiple tiles of confocal images were stitched to acquire a high-resolution image of a complete granule cell to enable a seamless analysis of dendritic protrusions along dendrites between the cell soma and outer tips of the dendritic tree. Counting and measurement of the tip sizes of small dendritic protrusions was performed using a self-developed plugin for ImageJ (for details see methods). Scale bars in panel **(B)** 100 μm, **(C)** 25 μm, **(C,** insert): 5 μm and (**D**, insert) 2 μm. GCL, granule cell layer; infra, infrapyramidal blade; ML, molecular layer; supra, suprapyramidal blade.

### 2.4 Fixation and immunohistochemistry

Filters containing OTCs were fixed either at 12 (*n* = 4), 14 (*n* = 3), 16 (*n* = 4), 18 (*n* = 3), 20 (*n* = 3) or 35 (*n* = 3) dpi with a PBS-based fixation solution containing 4% paraformaldehyde (PFA) and 4% sucrose. After 1 h of incubation at room temperature (RT), the fixative was changed to a 2% PFA/30% sucrose-containing solution. Cultures were incubated 24 h at 4°C and were washed 4 times with 0.1 M tris buffered saline containing 0.01% sodium azide (0.01 M TBS+0.01% NaN_3_) before being detached from the filters and placed into a 24-well-plate for further processing. Apart from pbGCs, retroviral labeling also marks newborn glial cells which, especially at early time points, can be confused with pbGCs. To correctly identify pbGCs at 12–20 dpi, we performed immunohistochemical staining for the granule cell marker Prox-1 in combination with doublecortin (DCX). For signal enhancement, we additionally stained all cultures for green fluorescent protein (GFP). The following staining protocol was applied at room temperature: Free-floating sections were washed 4 times with 0.1 M TBS and 0.01% NaN_3_. To prevent unspecific binding of antibodies, a blocking solution containing 5% Bovine serum albumin (BSA), 0.25% Triton X-100, and 0.01% NaN_3_ in 0.1 M TBS was applied for 1 h. Thereafter, sections were incubated with a primary antibody solution containing 2% BSA and 0.25% Triton X-100 in 0.1 M TBS for 3 days. The following antibodies were used: anti-GFP (mouse, polyclonal, 1:500, Sigma-Aldrich, St. Louis, MO, USA), anti-DCX (goat, polyclonal, 1:500, Cat# sc-8066, Santa Cruz Biotechnology, Santa Cruz, CA, USA) and anti-Prox-1 (rabbit, polyclonal, 1:1000, Cat# ABN278, Millipore, Schwalbach, Germany). Subsequently, sections were washed 4 times with 0.1 M TBS+0.01% NaN_3_ before they were treated with fluorescent dye-conjugated secondary antibodies for 2 days. The following secondary antibodies were used: Alexa Fluor 488 donkey anti-mouse for all cultures, and additionally Alexa Fluor 568 donkey anti-goat and Alexa Fluor 633 donkey anti-rabbit antibodies for 12–20 dpi OTCs (all from Invitrogen, Waltham, MA, USA). Antibodies were diluted at a ratio of 1:1000 in a TBS-based solution containing 2% BSA, 0.5% Triton X-100, and 0.01% NaN_3_. Finally, sections were washed 4 times with 0.1 M TBS containing 0.01% NaN_3_ and mounted with DAKO fluorescent mounting medium (Dako Cytomation, Carpinteria, CA, USA).

### 2.5 Confocal image acquisition and processing

To select suitable candidate cells for further analysis, 20 x and 40 x magnification image stacks were acquired with a confocal laser scanning microscope (Nikon D-Eclipse C2 controlled by Nikon EZ-C1 v3.60 software, equipped either with Nikon Plan Fluor 40 x, NA 1.3 or Plan Apo 20 ×, NA 0.75 objectives, all from Nikon Instruments, Amstelveen, the Netherlands). For the current study, we included only those pbGCs that were (a) located in the suprapyramidal blade or crest region of the DG, (b) exhibited granule cell morphology with an elaborated dendritic tree, (c) showed a high GFP-expression, (d) exhibited no or only slight overlap with other GFP-expressing cells, and (e) were double-immuno-positive for Prox-1 and DCX (12–20 dpi; Figures 1B, C). From cells that fulfilled these requirements, high resolution image stacks containing the complete dendritic tree were acquired (Table 2). Single dendritic segments were imaged with a 60 × magnification objective (Nikon Plan Ao VC, NA 1.4, Nikon Instruments, Amstelveen, the Netherlands) with 5 × field zoom at a resolution of 1024 * 1024 pixels and a z-step-size of 0.2 μm. Image stacks were stitched together using the ImageJ pairwise stitching tool. Stitched images were then deconvolved using Huygens professional software (V.23.04, Scientific Volume Imaging, Hilversum, The Netherlands) to optimize resolution and signal to noise ratio.

**TABLE 2:** Number (#) of analyzed animals, OTCs and cells.

Age	Animals (#)	OTCs (#)	Cells (#)
**12 dpi**	3	4	4
**14 dpi**	2	3	3
**16 dpi**	2	3	4
**18 dpi**	2	3	3
**20 dpi**	3	3	3
**35 dpi**	2	3	3

### 2.6 Analysis of dendritic trees

In order to differentiate between SDPs and short terminal branches, we consequently employed a cutoff at 5 μm for all protrusions that were counted and analyzed. Dendritic protrusions measuring more than 5 μm in length characteristically exhibited a larger diameter and, depending on the cell age, bore small protrusions at their shaft, and in some cases growth cones at their tip. They were thus considered as dendritic terminal branches. Dendritic protrusions measuring less than 5 μm on the other hand were much more abundant, thinner, and showed a high degree of structural heterogeneity. They were thus considered as SDPs (see below). Furthermore, studies in postnatal hippocampal cell cultures have described spine-like protrusions with a base-to-tip length ranging from 0.5 to 3 μm (Dailey and Smith, 1996) and dendritic filopodia, which typically did not exceed 5 μm in length, although some filopodia occasionally reached up to 10 μm (Ziv and Smith, 1996). This approach does not *per se* allow for the discrimination of an SDP from an emerging branch yet serves as a good approximation considering the much higher number of SDPs in comparison to small dendritic branches.

Apical dendrites of pbGCs were semi-automatically reconstructed using the SNT-plugin in ImageJ (Arshadi et al., 2021; Ferreira et al., 2014). Morphological data were acquired from reconstruction files using built-in-SNT-tools as well as self-written MATLAB scripts (Matlab R2024b; MathWorks Inc., Natick, MA, USA) based on methods from TREES toolbox^1^ (Cuntz et al., 2010, 2011).

### 2.7 Analysis of small dendritic protrusions (SDPs)

We aimed to perform SDP-analysis of complete dendritic trees of pre-selected cells (see section “2.5 Confocal image acquisition and processing”). However, some parts of the dendritic tree that (a) exhibited too little expression for analysis, (b) were overlayed by other GFP positive cells or dendrites, and (c) showed stitching artefacts were excluded from analysis. On average, we examined ∼80% ( ± 0.18 or 69–95%) of each cell’s total dendritic length (TDL). For numbers of analyzed neurons, dendritic segments and protrusions see Table 3. Analysis of SDPs was performed using a self-written ImageJ-plugin (SpineAnalyzerJ)^2^. After the definition of dendritic segments, all SDPs were counted and according to their morphology assigned into two different classes. Protrusions possessing a clearly recognizable bulbous head and a thin neck can be considered as morphological correlates for glutamatergic synapses (Harris et al., 1992; Papa et al., 1995; Toni et al., 2007) and were therefore referred to as “spines.” Protrusions without head represented mainly filopodia, but also irregularly shaped protrusions and presumably emerging branches. We therefore termed them as “other protrusions.” The tip size of each protrusion was determined by circumscription of its distal end (see inset of Figure 1D). Data acquisition for the SDP Sholl analysis of complete dendritic trees was performed by combining positional information of SDPs provided by SpineAnalyzerJ with information regarding the TDL per Sholl distance, calculated by a customized MATLAB script using methods from TREES toolbox (Cuntz et al., 2010). Relative SDP Sholl analysis was conducted by normalizing Sholl distances to the respective maximum dendritic span of individual cells. The resulting relative values were grouped into proximal (0–30%), middle (40–60%) and distal (70–90%) compartments for statistical analysis (Supplementary Figure 4) and covered approximately 88 ± 2 % of available dendrites in the proximal region, 90 ± 2 % in the medial region, and 81 ± 3 % in the distal region.

**TABLE 3:** Number (#) and length of analyzed neurons, dendritic segments, and protrusions.

Age	Cell	Segments analyzed for protrusions (#)	TDL (μm)	Segments analyzed for protrusions (μm)	Segments analyzed for protrusions (% of TDL)	Protrusions (#)	Spines (#)
**12 dpi**	1	43	890.05	615.03	69.10	32	12
**12 dpi**	2	20	637.02	535.67	84.09	77	53
**12 dpi**	3	32	903.24	681.81	75.49	26	15
**12 dpi**	4	20	736.46	568.77	77.23	51	28
**14 dpi**	5	84	1726.87	1543.52	89.38	202	77
**14 dpi**	6	66	1674.86	1443.61	86.19	99	49
**14 dpi**	7	52	1439.01	1187.37	82.51	176	122
**16 dpi**	8	48	1095.61	920.74	84.04	152	75
**16 dpi**	9	35	804.69	649.12	80.67	56	33
**16 dpi**	10	62	1572.62	1439.63	77.96	386	230
**16 dpi**	11	45	1846.53	1675.58	94.00	395	255
**18 dpi**	12	49	1558.76	1275.80	81.85	371	200
**18 dpi**	13	70	2006.87	1808.38	90.11	224	86
**18 dpi**	14	43	1697.37	1305.45	76.91	192	76
**20 dpi**	15	54	1685.73	1418.58	84.15	491	321
**20 dpi**	16	35	1243.48	874.05	70.29	53	34
**20 dpi**	17	82	1388.00	1304.48	93.98	398	242
**35 dpi**	18	25	1546.90	1474.91	95.35	2092	1075
**35 dpi**	19	40	1846.37	1738.49	94.16	1871	874
**35 dpi**	20	32	1767.21	1608.48	91.02	2226	1494

In addition, SDP analysis was conducted in a layer-specific way. For this purpose, the Euclidean distance from the soma to the border between the GCL and inner molecular layer (IML) was determined for each cell (see Figure 2A). All dendritic segments that extended beyond this boundary within a range of 40 μm in relation to the soma were assigned to the IML. All segments that extended further were assigned to the middle or outer molecular layer (MML, OML). The distance for the IML-MML-border to the GCL-IML-border was approximated and set to 40 μm based on anterograde tracing (Supplementary Figure 1).

**FIGURE 2:**
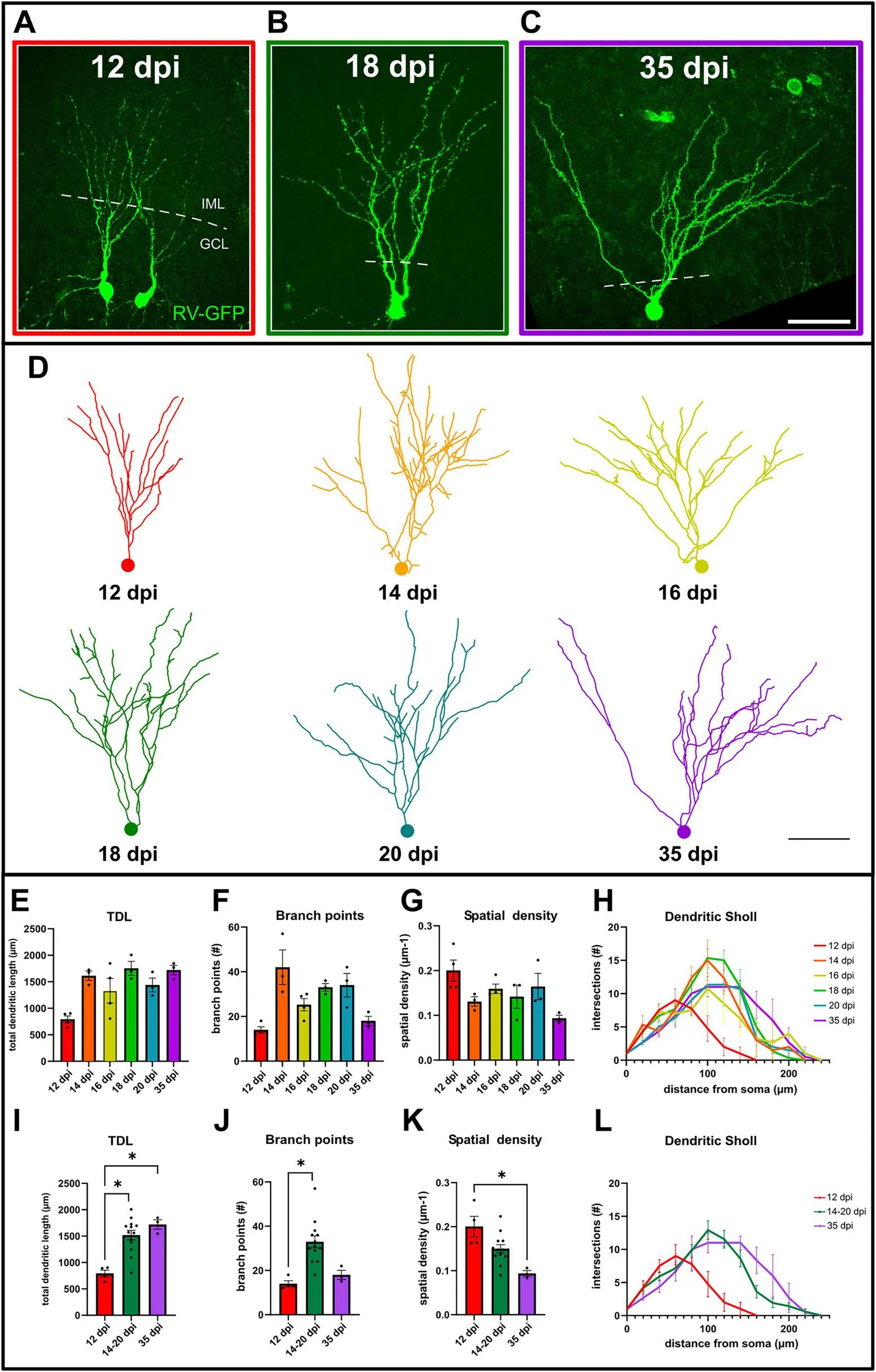
Dendritic maturation of postnatally-born hippocampal granule cells (pbGCs). **(A–C)** Z-projections of confocal image stacks presenting pbGCs at 12, 18, and 35 dpi. Dashed lines mark borders between the granule cell layer (GCL) and inner molecular layer (IML). **(D)** Examples for dendritic reconstructions of pbGCs at different cell ages (12–35 dpi). Structural analysis was performed to obtain the **(E)** total dendritic length (TDL), **(F)** number of branch points, **(G)** dendritic spatial density and **(H)** Sholl analysis for all individual time points. Data were pooled into 12, 14–20, and 35 dpi for statistical analysis **(I–L)**. For detailed statistical reporting see also Supplementary Tables 5–7. Number of analyzed cells: 12 and 16 dpi: *n* = 4; 14,18,20, and 35 dpi: *n* = 3. Data in panels **(E–L)** are presented as mean values with whiskers indicating SEM. (**I–K**) *P < 0.05, Kruskal-Wallis-test followed by Dunn’s multiple comparison test. Scale bars in panel **(C)** and **(D)** 50 μm. #, number.

### 2.8 Assessment of dentate gyrus (DG) connectivity at DIV 10

Local AAV-hSyn-ChR2-tdTomato-injections into the DG or the EC were performed after OTC preparation at DIV 0 to label mature EC neurons and their axonal projections toward the DG. Injections were carried out with the same parameters and setup as described for RV-injections (see above). After incubation for 10 days, DG connectivity was evaluated by live imaging of OTCs using a confocal laser scanning microscope (Fluorview 3000, Olympus) equipped with a 20 x objective (XLUM Plan FI 20x, NA 0.95, Olympus) as previously described (Greco et al., 2025). Confocal image stacks were acquired with a resolution of 1024 by 1024 pixels and a z-interval of 1 μm.

### 2.9 Statistical analysis and digital illustrations

Data was stored and managed in Excel 365 (Microsoft, Redmond, WA, USA) with the help of built-in-functions and customized python scripts. Statistical analysis and visualization of graphs was performed with GraphPad Prism (version 10.6.1, GraphPad Software, San Diego, CA, USA). Statistical comparisons were either calculated using the Kruskal-Wallis with Dunn’s multiple comparisons test (Figures 2I–L, 3B, E, H, K, M) or the Wilcoxon matched pairs signed rank test (Figures 4F, L). Significance level was set at *P* < 0.05. Group values are reported as means ± SEM. All statistical comparisons are based on neurons as biological units. Microscopic image stacks were edited with ImageJ version 1.54f (Schneider et al., 2012) for contrast, rotation, cropping, and z-projections. Figures were arranged and schematic drawings were created in Inkscape (version 13.2, Inkscape Project (2020). Inkscape. Retrieved from)^3^.

**FIGURE 3:**
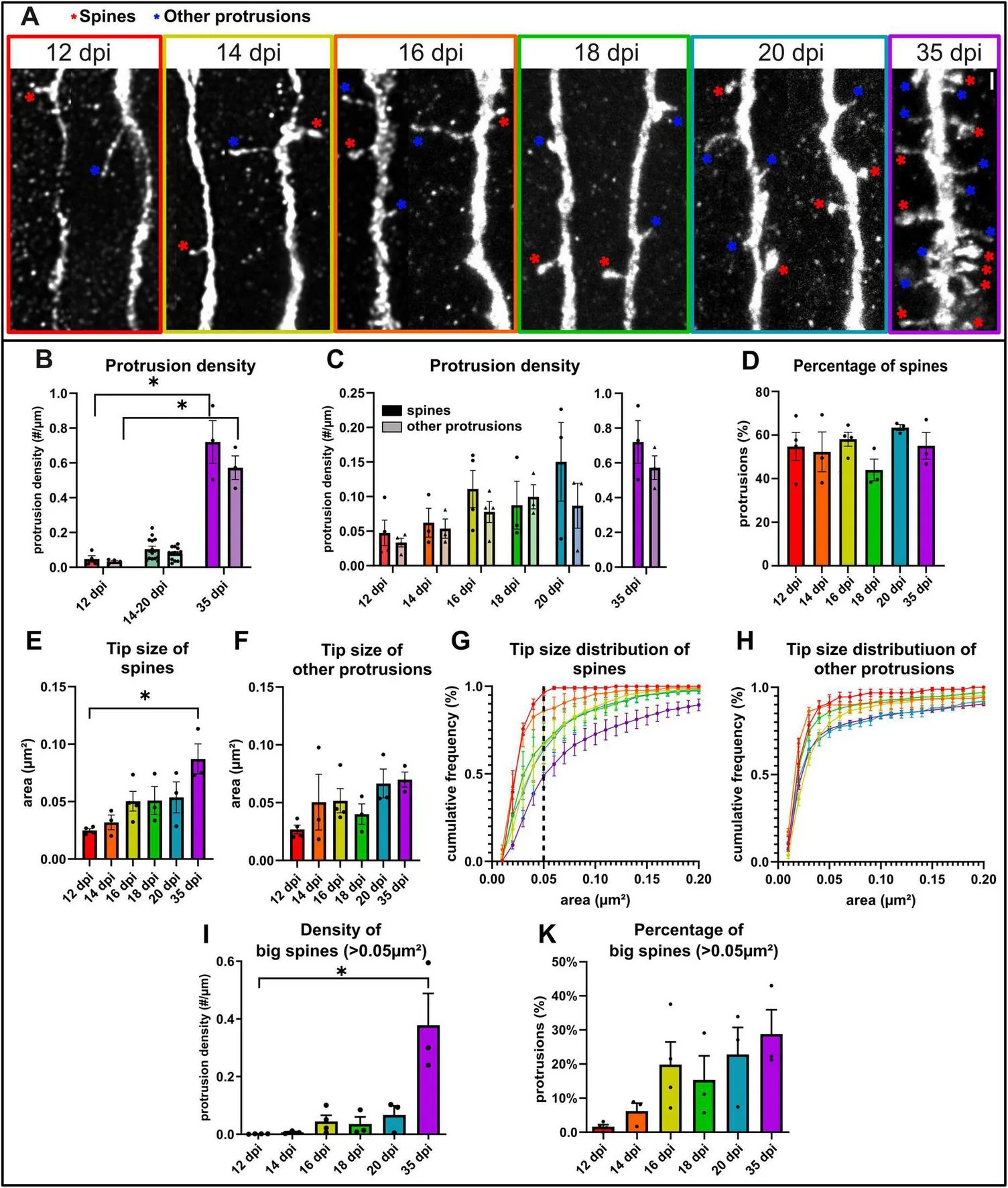
Small dendritic protrusions (SDPs) on postnatally-born granule cells (pbGCs) were present at 12 dpi and increased in number over time. **(A)** Representative examples of SDPs with head (referred to as spines, indicated by red asterisks) and protrusions without head (designated as other protrusions, indicated by blue asterisks) at all studied time points. Density of spines and other protrusions for pooled **(B)** and individual time points **(C).** **(D)** Percentage of spines in relation to all SDPs. Tip size of spines **(E)** and other protrusions **(F)** at all individual time points and as cumulative frequency distributions **(G, H)**. Dashed line in **(G)** indicates the threshold of 0.05 μm^2^ used to identify big spines. Density of big spines with a measured tip size larger than 0.05 μm^2^ at individual time points **(I)** and as percentage in relation to all SDPs **(K)**. For detailed statistical reporting see also Supplementary Tables 8–12. Number of analyzed cells: 12 and 16 dpi: *n* = 4; 14,18, 20, and 35 dpi: *n* = 3. Data in panels **(B-K)** are presented as mean values with whiskers indicating SEM. (**B**,**E,I**) *P < 0.05, Kruskal-Wallis-test followed by Dunn’s multiple comparison test. Scale bar in panel **(A)** 0.5 μm.

**FIGURE 4:**
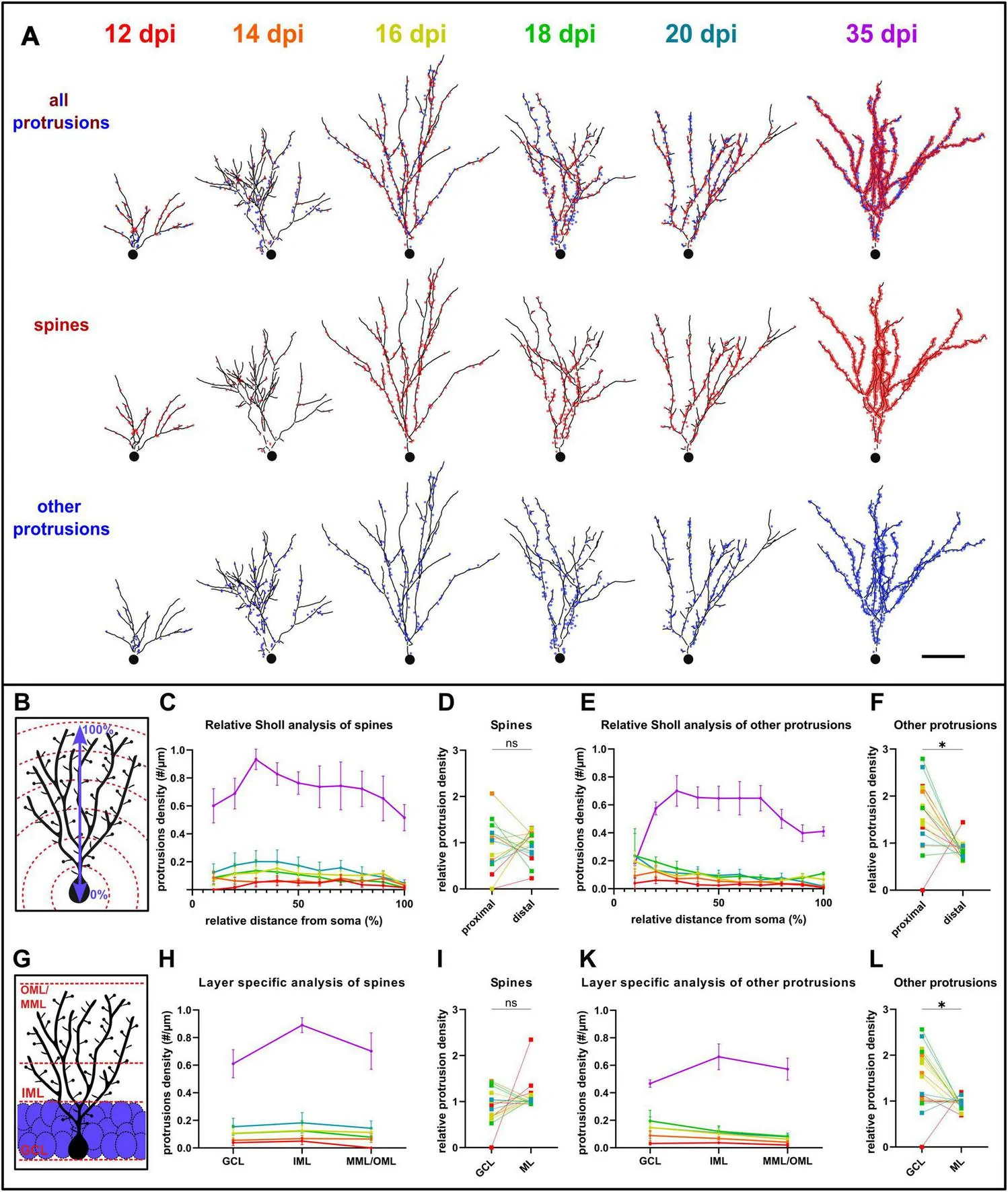
Spines were equally distributed throughout the dendritic tree whereas other protrusions follow a proximo-distal gradient and were more abundant in the granule cell layer (GCL) at early time points. **(A)** Distribution of spines and other protrusions in the dendritic tree of representative examples at all studied time points. **(B)** Relative Sholl analysis of small dendritic protrusions (SDPs) was performed to obtain densities in relation to their position on the dendritic tree. Relative distribution of spines **(C)** and other protrusions **(E)**. Densities of spines **(D)** and other protrusions **(F)** of proximal and distal parts of the dendritic tree were normalized to the mean density of individual cells (time points included: 12-20 dpi). **(G)** SDP distributions were assessed in a layer-specific approach subdivided into the granule cell layer (GCL), inner molecular layer (IML) as well as middle and outer molecular layer (MML/OML). Additionally, data of the IML, MML, and OML were combined into the molecular layer (ML). Layer-specific densities of spines **(H)** and other protrusions **(K)**. Normalized data of spines **(I)** and other protrusions **(L)** for dendritic parts in the GCL and ML. Number of analyzed cells: 12 and 16 dpi: *n* = 4; 14, 18, 20, and 35 dpi: *n* = 3. Data in panels **(C–F,H–L)** are presented as mean values with whiskers indicating SEM. **(F**,L)*P < 0.05, Wilcoxon matched-pairs signed-rank test. Scale bar in panel **(A)** 50 μm.

## 3 Results

The goal of the study was to analyze the appearance of SDPs, including spines, in developing dendritic trees of pbGCs which were labeled with a retrovirus encoding for GFP under the CAG promotor (Van Praag et al., 2002) by local injection in the DG subgranular zone of OTCs derived from neonatal (P4/5) rats (Figure 1).

### 3.1 General dendritic maturation of pbGCs in OTCs

We focused our characterization of the general dendritic structure of immature pbGCs at 12–20 days post RV injection (dpi), the timespan of early spinogenesis as well as major enlargement and reorganization of the dendritic tree within the ML (Radic et al., 2017). The morphology of those immature pbGCs was then compared to pbGCs at 35 dpi, a timepoint at which the dendritic trees of pbGCs are considered structurally maturated (Radic et al., 2017). RV-labeled pbGCs attained a sufficient GFP-expression throughout large parts of their dendritic tree by 12 dpi, a requirement for unambiguous detection of SDPs and therefore the start of our investigation. In addition, this timepoint coincides with the establishment of general synaptic connectivity of the DG in OTCs derived from postnatal rats, including incoming entorhinal afferents (Kluge et al., 1998; Li et al., 1994). We confirmed this by performing local AAV-hSyn-injections in the DG or EC to label mature neurons, and subsequent examination of local and entorhinal projections at DIV 10 (Supplementary Figure 1).

At 12 dpi, cell bodies of pbGCs were positioned in the lower third of the GCL (Figure 2A). At this stage, cells displayed a developed apical dendritic tree (Figures 2A, D) while still retaining basal dendrites and dendritic varicosities, characteristic features for immature granule cells (Jones et al., 2003; Lübbers and Frotscher, 1988). The TDL was still small (Figures 2E, I), dendritic trees had few branch-points (Figures 2F, K), a high spatial density (Figures 2G, L) and Sholl analyses revealed that the dendritic arbor extended up to 150 μm from the soma (Figures 2H, M). Only one out of four analyzed cells reached through the GCL into the IML but did not extend into the MML and OML. Taken together, pbGCs at 12 dpi showed a characteristic morphology of immature growing neurons typical for the second week of their development (Radic et al., 2017).

Cells at later time points (14–20 dpi) reached their maximum TDL and exhibited an enlarged, more elaborated dendritic tree (Figures 2B, D) across all parts of the ML, with some dendrites reaching the hippocampal fissure. In comparison to 12 dpi, pbGCs at 14–20 dpi showed a significantly larger TDL (12 dpi: 791.7 ± 63.96 μm, 14–20 dpi: 1518 ± 90.07 μm; Figures 2E, I and Supplementary Table 5) and a higher number of branch points (12 dpi: 14.00 ± 1.35, 14–20 dpi: 32.92 ± 2.69; Figures 2F,K and Supplementary Table 6). Sholl analysis revealed an increase in dendritic complexity and a shift of the maximum complexity toward more distal parts of the dendritic tree (Figures 2H, M). Altogether, these findings indicate an ongoing dendritic reorganization which is characteristic for the third week of dendritic development (Radic et al., 2017).

At 35 dpi, somata of pbGCs were localized in middle parts of the GCL and the cell morphology showed major characteristics of mature GCs (Figures 2C, D). While the TDL only moderately increased from 14 to 20 dpi in comparison to 35 dpi (14–20 dpi: 1518 ± 90.07 μm, 35 dpi: 1720 ± 89.59 μm; Figures 2E, I and Supplementary Table 5), pbGCs at this stage showed reduced numbers of branch points (35 dpi: 18.00 ± 2.08; Figures 2F, K and Supplementary Table 6) and a significantly lower spatial density when compared to 12 dpi (12 dpi: 0.20 ± 0.02 μm^–1^, 35 dpi: 0.093 ± 0.01 μm^–1^; Figures 2G, L and Supplementary Table 7), consistent with the state of structurally mature GCs after the phase of dendritic refinement present within the fourth week of development (Radic et al., 2017).

Overall, these results indicate a cell age-related phasic growth and maturation of pbGCs in OTCs as already reported in earlier studies (Radic et al., 2017). Therefore, we considered our data set suitable for a detailed analysis of spine formation.

### 3.2 Classification of small dendritic protrusions (SDPs)

Our dataset of high-magnification z-stacks of complete dendritic trees of individual pbGCs (see methods) enabled us to perform detailed analysis on their coverage of dendritic protrusions. All protrusions that were smaller than 5 μm in length were registered as SDPs. Typical for young neurons, early SDPs had a high variety of sizes and shapes (see Figure 3A; Dailey and Smith, 1996; Jones et al., 2003) that could be either dendritic spines, filopodia, dendritic branchlets, or growth cone sprouts. In young neurons, the majority of SDPs exhibiting a bulbous head and small neck form functional glutamatergic synapses (Harris et al., 1992; Papa et al., 1995; Toni et al., 2007). Consistent with the literature, we classified SDPs into two classes: small protrusions that contained a clearly discernible head and neck and therefore exhibited morphological characteristics associated with glutamatergic synapses were classified as “spines” and other small protrusions of various shapes of filiform or more complex character (Dailey and Smith, 1996) that were likely to be rather growth- than activity-driven (Dailey and Smith, 1996; Hossain et al., 2012; Niell et al., 2004; Vaughn et al., 1974). The latter were labeled as “other protrusions.” In addition to this morphological classification, we measured the tip size of each SDP (see insert Figure 1D). Previous ultrastructural reports on *in vivo* abGCs revealed that only protrusions with a bulbous head and a tip diameter larger than 0.25 μm (corresponds to an area of about 0.0491 μm^2^) featured morphological characteristics of mature excitatory synapses, whereas protrusions with a smaller tip diameter were devoid of synapses at their tip (Toni et al., 2007). We therefore classified protrusions with a prominent head that exhibited a size of more than 0.05 μm^2^ as “big spines.” We analyzed 937 dendritic segments from 20 cells in total, covering ∼80% ( ± 0.18, 69–95%) of their TDL.

### 3.3 Spines and other protrusions are present from 12 dpi on throughout the dendritic tree of pbGCs and increase in number over time

At 12 dpi, we found ∼47 (range 26–77) SDPs in all four cells that were analyzed, located across all parts of the dendritic tree (Supplementary Figure 2 and Figure 4A), resulting in a very low total protrusion density of 0.08 ± 0.05 μm^–1^, with a spine density of 0.05 ± 0.02 μm^–1^, and a density of other protrusions of 0.03 ± 0.01 μm^–1^; Figures 3B, C and Supplementary Table 8). Interestingly, about half of all SDPs (54.7 ± 13.0%, Figure 3C) exhibited a head and were therefore classified as spines. However, most of those spines were small (96.6 ± 3.6%) and exhibited a tip size smaller than 0.05 μm^2^ (Figure 3G). Accordingly, virtually none of the protrusions fulfilled the morphological criteria of a big, “mature” spine (Figures 3I, K).

Analysis of the density of SDPs over time revealed a trend indicating a continuous increase in both spines and other protrusions from 12 to 20 dpi (densities of spines 12 dpi: 0.05 ± 0.02 μm^–1^, 20 dpi: 0.15 ± 0.06 μm^–1^, densities of other protrusions 12 dpi: 0.03 ± 0.01 μm^–1^, 20 dpi: 0.09 ± 0.03 μm^–1^; Figure 3C) resulting in a total protrusion density of 0.24 ± 0.15 μm^–1^ at 20 dpi. Interestingly, the proportion of spines didn’t change much over time and stayed relatively consistent at about 50–60% of all protrusions (Figure 3D), but when looking at spine dimensions, we found a moderate but constant increase of their tip size from 12 to 35 dpi (Figure 3E and Supplementary Table 9).

In order to compare the phase of early spinogenesis (12 – 20 dpi) to a more advanced stage of development, we analyzed pbGCs at 35 dpi which are considered mature and already functionally integrated within the hippocampal network (Radic et al., 2017), similar to abGCs of the same age *in vivo* (Espósito et al., 2005; Jungenitz et al., 2018; Toni et al., 2007; Zhao et al., 2006). We observed an increase in the protrusion density from 12 to 35 dpi that included spines (12 dpi: 0.05 ± 0.02 μm^–1^, 35 dpi: 0.72 ± 0.12 μm^–1^) as well as other protrusions (12 dpi: 0.03 ± 0.01 μm^–1^, 35 dpi: 0.57 ± 0.07 μm^–1^) which was, in both cases, approximately four times higher compared to cells at 20 dpi (Figures 3B, C and Supplementary Table 8). This progression was accompanied by an increase of the spine tip size (12 dpi: 0.025 ± 0.002 μm^2^, 35 dpi: 0.09 ± 0.01 μm^2^; Figure 3E and Supplementary Table 9) and the size of other protrusions (12 dpi: 0.027 ± 0.004 μm^2^, 35 dpi: 0.07 ± 0.01 μm^2^; Figure 3F and Supplementary Table 10). Using a frequency analysis (Figures 3G, H), we determined the density and percentage of spines with head areas larger than 0.05 μm^2^ (designated as “big spines”), revealing that pbGCs at 12 dpi were almost completely devoid of big spines (0.0013 ± 0.0004 μm^–1^), cells between 16 and 20 dpi exhibited a low density (16 dpi: 0.05 ± 0.02 μm^–1^), and substantial number of big spines only emerged at 35 dpi (0.38 ± 0.11 μm^–1^; Figure 3I and Supplementary Table 11). The proportion of big spines increased steadily from 12 to 35 dpi (12 dpi: 1.6 ± 0.7 %, 16–20 dpi: 16.4 ± 3.3 %, 35 dpi: 28.8 ± 7.1 %; Figure 3K and Supplementary Table 12).

Taken together, our data show that spines and other protrusions could be detected from 12 dpi onward and exhibited a steady increase in numbers within the third week of development. While the size of other protrusions remained relatively constant, spine sizes increased over time with first big spines occurring in small numbers from 16 dpi onward which may indicate the onset of synaptic maturation and the establishment of the first functional synaptic contacts which would be consistent with prior ultrastructural studies (Toni et al., 2007).

### 3.4 Spines are evenly distributed throughout the dendritic tree whereas other protrusions follow a proximo-distal gradient and are preferentially located in the GCL

Next, we analyzed the spatial distribution of dendritic protrusions at individual cells (Figures 4A, 5). In general, we detected a noticeable increase in the number of SDPs throughout all parts of the dendritic tree over time. At early time points between 12 and 14 dpi, the distribution of SDPs was not consistent throughout the dendritic tree, and some dendritic segments showed clusters of SDPs whereas other parts were completely devoid of protrusions. To quantify the distribution of SDPs in relation to their distance to the soma, Sholl analyses for different protrusion types were performed for all time points (Supplementary Figure 3A–C). For a better comparison between cells of different ages and to account for morphological variabilities, we normalized the Sholl intersections relatively to the maximal dendritic span of each individual cell (Figure 4B). This relative Sholl analysis revealed a quite uniform density of spines and other protrusions in most parts along the dendritic tree for pbGCs of all observed timepoints (Figures 4C, E). Additionally, we pooled data from 12 to 20 dpi cells and normalized their average protrusion density of proximal (0–30% of maximal dendritic span) and distal segments (70–90% of maximal dendritic span) with the mean protrusion density of individual cells. Spines did not show prevalence for proximal or distal parts of the dendritic tree (Figure 4D), whereas other protrusions were found more frequently in proximal vicinity to the soma (proximal: 1.61 ± 0.17, distal: 0.83 ± 0.05; Figure 4F). Interestingly, there were no differences in the tip size of spines related to the position (Supplementary Figure 3D), nor a selective distribution of “mature” big spines (Supplementary Figure 3E).

**FIGURE 5:**
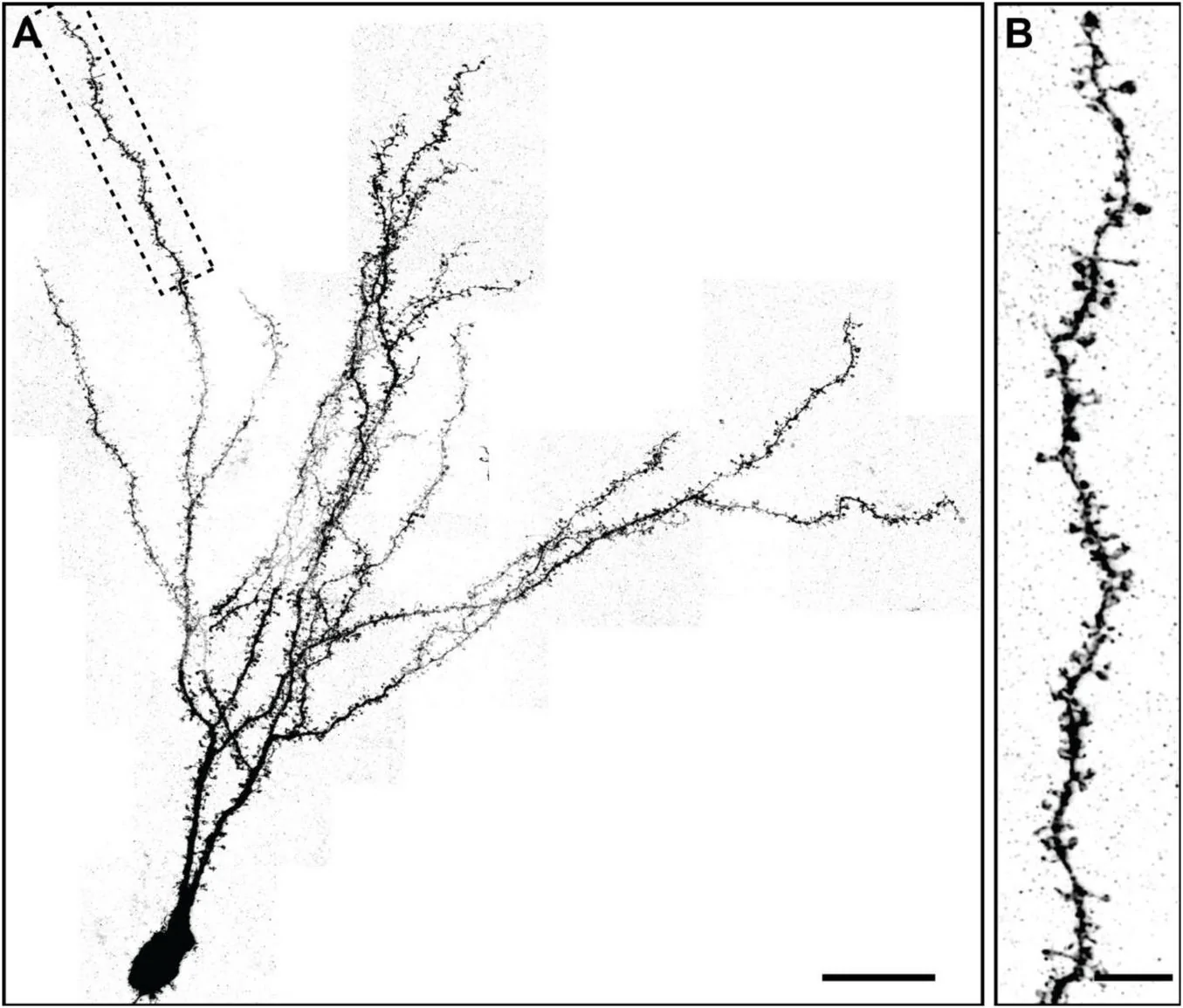
Stitched high-resolution image of a dendritic tree at 35 dpi. (**A**) Individual dendritic segments were imaged with a 60 × magnification objective. The acquired image stacks were stitched and deconvolved before further processing. The depicted image was optimized by removing obstructing structures not belonging to the cell (e.g., GFP-labeled astrocytes), applying a median filter, and a maximum z-projection was generated. The grayscale image was inverted to enhance the visual contrast. (**B**) Higher magnification of a selected dendritic segment. Note the rich morphological variability of dendritic protrusions. Scale bars in panel **(A)** 25 μm and **(B)** 5 μm.

PbGCs in the OTC grow and integrate into the DG which receives its input in a lamina-specific way, which is already established at DIV 10 (Supplementary Figure 1; Amaral et al., 2007; Hildebrandt-Einfeldt et al., 2021; Kluge et al., 1998; Li et al., 1994; Radic et al., 2017). To account for this fact, we assessed protrusion densities for pbGCs in the GCL as well as the IML, MML, and OML (Figure 4G). We detected a high number of SDPs that were located in the GCL at all time points (Figures 4H, K). Interestingly, we didn’t find large differences in spine densities between the different sublayers of the DG, even when comparing relative protrusion densities from the GCL to the ML (Figure 4I). When we examined the density of other protrusions, we discovered that the proximo-distal gradient described above was mainly caused by a preferential concentration of these protrusions within the GCL (Figures 4K, L).

In conclusion, spines, including big spines, were evenly distributed throughout the dendritic tree in all layers of the DG during the period of early spinogenesis whereas other protrusions showed a preference to proximal parts of the dendritic tree, especially the GCL. As no distinct layer exhibited a concentration of spines, we found no direct evidence for layer-specific influence on the establishment of synaptic contacts.

## 4 Discussion

In this study, we were primarily focused on the dendritic growth and the onset of spinogenesis during the early stages of pbGC development using fully reconstructed and annotated dendritic trees in rat OTCs, with a particular focus on the laminar organization of the molecular layer. Our findings are generally corroborating previous reports on spinogenesis in developmental and adult-born GCs, both *in vitro* and *in vivo*, reporting an early onset of spines and suggesting a more or less even spine distribution. However, studies so far have been based on spine analysis of selected dendritic segments, but not complete trees, leaving open the possibility of layer-specific changes in spine distribution. This is the first study of spinogenesis in complete dendritic trees of young pbGCs, thus adding a new level of precision. Spines were evenly distributed throughout all parts of the dendritic tree as early as from 12 dpi on with no preference for any layer of the DG, whereas other protrusions exhibited a proximo-distal distribution and were more likely located in the GCL than in the ML. In conclusion, pbGCs emerging from neural stem cells in the SGZ are growing in an existing neural network and while we cannot fully estimate, nor exclude the influence of extrinsic cues, our data suggests that dendritic structures are established with a strong contribution from intrinsic developmental programs (Figure 6).

**FIGURE 6:**
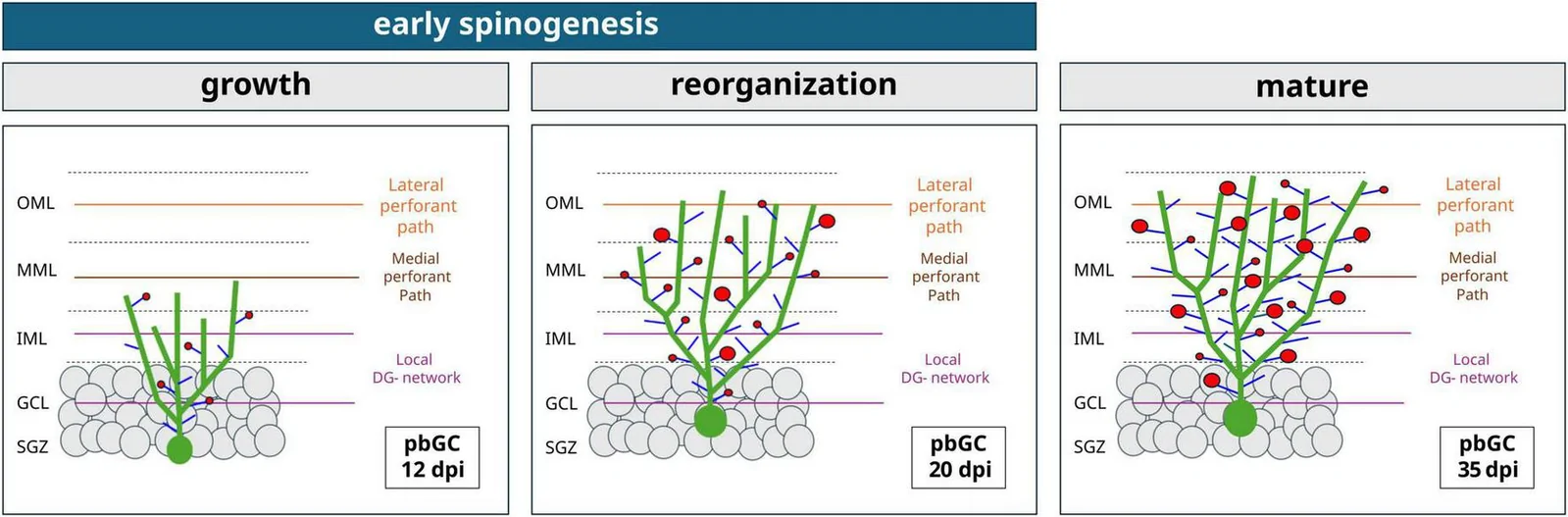
Spinogenesis in postnatally-born hippocampal granule cells (pbGCs). PbGCs emerging from the subgranular zone (SGZ) of the dentate gyrus (DG) develop and integrate into an existing neural network which is organized in a layer-specific way in organotypic entorhino-hippocampal slice cultures (OTCs). While local DG afferents mainly target the granule cell layer (GCL) and inner molecular layer (IML), projections arising from the medial and lateral entorhinal cortex pass through the medial and lateral perforant path to terminate at the middle and outer molecular layer (MML/OML). Structural maturation of pbGCs extends over several weeks. Early spinogenesis of pbGCs takes place during the time of growth and reorganization of the dendritic tree. First spine-like small protrusions with head (indicated with a red dot) and other small protrusions without head (indicated in blue) can be observed from 12 days post retroviral injection (dpi) and increase in numbers over time. Between 12–35 dpi, pbGCs display a progressive increase in spine size. While spines are evenly distributed throughout the dendritic tree irrespective of the layer within the DG, other protrusions are preferentially located at proximal parts in the GCL. The uniform distribution of spines throughout the dendritic arbor during dendritic maturation and reorganization suggests that spinogenesis follows an intrinsic cellular program rather than being driven by differential spine formation in response to extrinsic, layer-specific cues.

Previous research covering multiple animal models revealed that neural stem cells, even when implanted into areas of damaged neuronal tissue, show regular dendritic differentiation, spinogenesis, and synaptic integration (Berg et al., 2024; Muñetón-Gómez et al., 2012; Tønnesen et al., 2011). The ability of these cells to develop and form synaptic contacts without the need for specific afferent network activation holds considerable potential in future treatment of neurodegenerative diseases such as Parkinson’s Disease, Huntington’s Disease, and stroke (Holley et al., 2023; Sawamoto et al., 2025; Tabar et al., 2025; Weber et al., 2025).

### 4.1 Dendritic development of pbGCs in the OTC

Proper development of an age-appropriate dendritic tree is a prerequisite for a successful synaptic integration, survival, and functioning of new neurons, as studied in many details in abGCs (Denoth-Lippuner and Jessberger, 2021; Kempermann et al., 2015). Therefore, we analyzed complete dendritic reconstructions of pbGCs between 12 and 35 dpi for various morphological parameters including TDL, branch points, length of terminal segments, and Sholl analysis. Our results are in line with previous reports on the structural development of pbGCs *in vitro* (Kamada et al., 2004; Namba et al., 2011; Radic et al., 2017; Raineteau et al., 2006, 2004), and show a general comparability to the structural development of abGCs *in vivo* (Beining et al., 2017; Cole et al., 2020; Espósito et al., 2005; Zhao et al., 2006). The development of dendrites determines which layer-specific inputs in the DG pbGCs can connect to at a given stage of their development. At 12 dpi, most pbGCs extend their processes through the GCL into the IML and only rarely reach the MML, which means that they are limited to local DG-input. From 14 dpi onward, pbGCs also reach the MML and in some cases even the OML and thus can also receive input from EC-fiber-projections.

These findings are in line with morphological, electrophysiological, and retrograde tracing studies in adult rodents performed *in vivo* which found that during the second week of maturation, abGCs mainly receive input from local GABAergic interneurons and mossy cells, whereas afferent input from the EC becomes increasingly important during the third week of maturation (Deshpande et al., 2013; Espósito et al., 2005; Ge et al., 2006; Sah et al., 2017; Vivar et al., 2012).

### 4.2 Small dendritic protrusions (SDPs) can be observed at 12 dpi

12 dpi was the earliest time point at which we could observe a low number of SDPs, spines and other protrusions, that were distributed throughout the entire dendritic tree. To our knowledge, this represents the earliest time point reported so far in postnatal rat OTCs and precedes our previous findings, in which the first presence of spine-like structures in middle parts of the dendritic tree was reported at 14 dpi (Radic et al., 2017). The results of our study are well in line with *in vivo* data from postnatal mice, which revealed that spinogenesis in new GCs begins as early as 12 dpi, whereas in adult animals it is delayed until 16 dpi (Zhao et al., 2006). Similar findings were reported by Ohkawa et al., 2012, who investigated the onset of spine development in the middle and outer parts of the dendritic tree of new GCs in an *in vivo* study using adult rats. They found no dendritic spines at 12 dpi and the first appearance of spines beginning at 16 dpi (Ohkawa et al., 2012). In general, adult maturation of GCs is significantly delayed compared to the embryonic and early postnatal period (Cahill et al., 2017; Overstreet-Wadiche et al., 2006). Therefore, our findings of an early onset of spinogenesis in pbGCs during the second week of maturation in postnatal OTCs closely mirrors the *in vivo* condition and confirms previous findings indicating that spine formation begins earlier in neonatal animals than in adults.

### 4.3 Mild increase in protrusion density from 12 to 20 dpi

We observed only a mild increase in the density of protrusions during the early phase of spine development from 12 dpi (0.08 μm^–1^) to 20 dpi (0.24 μm^–1^), resulting in a relatively low density compared to the maturated stage at 35 dpi (1.3 μm^–1^). The delayed progression of spinogenesis in our data could be explained due to differences in the amount of general network activity which is supposedly lower in the OTC compared to *in vivo* (Prang et al., 2003). During the first weeks after preparation from postnatal tissue, OTCs essentially remain in an immature state despite local DG and EC connectivity being established (Bahr, 1995; Dailey and Smith, 1996). Nevertheless, spine density further increased in our study until 35 dpi, indicating that maturation of pbGCs was still in progress and spine density could still increase with time, as this was shown for abGCs *in vivo* at later time points up to 77 dpi or even longer (Cole et al., 2020; Jungenitz et al., 2018).

In summary, SDPs existed from 12 dpi on, and only slowly increased with time. However, big spines with tip sizes larger than 0.05 μm^2^ started to emerge at 16 dpi which is in line with previous *in vivo* research on adult animals (Cole et al., 2020; Ohkawa et al., 2012). Therefore, our results further substantiate the general start of considerable numbers of spines with large, bulbous heads at 16 dpi, a timepoint when dendritic dynamics in pbGCs reach their maximum. This indicates that during the third week of development spinogenesis and dendritic reorganization are interrelated processes.

### 4.4 Early spinogenesis occurs in all parts of the dendritic tree

In our study, we sought to determine differences in the coverage of SDPs along different parts of the dendritic tree. We found that at early stages (12–20 dpi), other small protrusions were more abundant in proximal compared to distal parts of the dendritic tree and preferentially located within the GCL. Whereas GCs receive strong inhibitory input from surrounding DG interneurons, excitatory input in this cell layer is sparse (Amaral et al., 2007). However, SDPs have been described to be present in a low number on proximal dendrites of abGCs (Puhahn-Schmeiser et al., 2022). During early brain development, proximal filiform processes are thought to play a role in migratory processes (reviewed in Wit and Hiesinger, 2023). Therefore, other protrusions seem to be associated with growth and migration rather than activity-related cellular processes. On the other hand, spines, including big spines, were evenly distributed throughout the dendritic tree, suggesting that early synaptic integration does not preferentially target any specific sublayer of the molecular layer. Previous literature revealed that a conditional knock-down of NMDA receptors in abGCs did not significantly affect the formation of spines in these cells but it did reduce their survival, indicating that spinogenesis in new GCs is largely independent of afferent synaptic input (Tashiro et al., 2006). This is supported by further studies investigating early synapse formation in the brain reporting that an early onset of spine development does not depend on neurotransmitter signaling (Sando et al., 2017; Sigler et al., 2017; Verhage et al., 2000), suggesting that internal processes govern spinogenesis of immature neurons. Indeed, several genes and molecular pathways have been found to influence this process in new GCs (Bosch et al., 2016; Duan et al., 2014; Llorens-Martín et al., 2013). In addition to internal factors, spinogenesis in abGCs has been found to be regulated by surrounding astrocytes (Sultan et al., 2015) as well as neurotrophic factors and hormones (Bonafina et al., 2019; Robert et al., 2025; Zhang et al., 2016). Therefore, in accordance with previous literature on abGCs, our findings support the idea that early spinogenesis in new GCs is predominantly driven by activity-independent mechanisms. Additionally, the uniform distribution of dendritic spines observed in pbGCs likely reflects their relatively immature stage, which may progressively evolve into the laminar-specific pattern observed in abGCs *in vivo* (Jungenitz et al., 2018).

Despite the early onset of spinogenesis during the third week of maturation, behavioral studies suggest that abGCs start to contribute to hippocampus-dependent tasks during the fourth week of development (Kee et al., 2007; McHugh et al., 2022) when they show a high degree of excitability and synaptic plasticity (Ge et al., 2006; Schmidt-Hieber et al., 2004; Vivar et al., 2012) and exhibit a gradual response to homo- and heterosynaptic long term potentiation and depression (Jungenitz et al., 2018). Thus, in a more general concept, development of the dendritic tree and its functional reorganization toward a mature state would occur during the first 3 weeks of maturation. Starting at the fourth week of maturation, new GCs would then enter a so called “critical phase” during which dendritic refinement, together with enhanced spinogenesis, would be under regulatory influence of layer-specific afferent activity, leading to specific connectivity and differential synaptic weights established with mechanisms of Hebbian synaptic plasticity such as long term potentiation and depression (Ge et al., 2006; Schmidt-Hieber et al., 2004; for reviews see: Denoth-Lippuner and Jessberger, 2021; Toni and Schinder, 2016).

### 4.5 Dendritic spines on pbGCs can be observed in the GCL in OTCs

Interestingly, dendritic segments located in the GCL and ML exhibited SDPs with heads in comparable numbers at all observed timepoints (12–20, 35 dpi). Spines have been described to be present on dendrites of GCs in in the GCL of adult mice in vivo, though to a considerably lower amount when compared to the ML (Puhahn-Schmeiser et al., 2022). However, the substantial numbers of spines in the GCL we found in our study might be due to the lower cell density in the GCL of OTCs (Sadgrove et al., 2006), allowing for more synapses to be formed.

### 4.6 Role of SDPs in general

We found a high variability in the morphology of SDPs in pbGCs which is typical for immature GCs (Jones et al., 2003). Ultrastructural studies involving abGCs revealed that spines with a prominent head (diameter larger than 0.25 μm, corresponds to an area of 0.05 μm^2^) were a correlate for glutamatergic synapses whereas smaller protrusions (diameter smaller than 0.25 μm, corresponds to 0.05 μm^2^) were devoid of synapses (Toni et al., 2007). Combined with our observation that spines with heads increased in number and size these findings suggest an increase in the number of glutamatergic synapses on new GCs between 12–35 dpi. Previous literature suggests that protrusions of immature neurons are very plastic, can rapidly change their morphology, and even interconvert between different types of spines within minutes (Dailey and Smith, 1996; Dunaevsky et al., 1999; Parnass et al., 2000). Over time, there is a shift from filiform and complex shaped protrusions toward more mature spines with large, bulbous heads. This shift is also accompanied by a general stabilization of dendritic spines (Dailey and Smith, 1996; Toni et al., 2007; Zhao et al., 2006). Therefore, irregular protrusions and filopodia might act as proto-spines during the establishment of synaptic contacts. Nevertheless, in our study, the rate of irregular protrusion without heads did not change over time and remained high even at 35 dpi. Our findings could be explained by a prolonged immature state of our OTCs with a lower connectivity which would account for more structural plasticity and less stabilization (Bahr, 1995; Dailey and Smith, 1996).

### 4.7 Limitations and outlook

Our study has several limitations that should be acknowledged. First, the number of analyzed neurons at individual time points was relatively small. Therefore, neurons between 14 and 20 dpi were pooled into a single group for statistical testing, allowing robust comparisons primarily between younger (12 dpi) and more mature pbGCs (35 dpi), while limiting analysis within the pooled intermediate cohort. Second, due to low GFP expression levels, we started our analysis at 12 dpi, when we could already observe SDPs. Therefore, our method doesn’t allow any assumption on the coverage of SDPs at earlier timepoints. Protrusions of immature neurons are very plastic and possess a variety of morphologies (see results). In order to get valuable insights about the distribution of small protrusions along the dendritic tree, we performed a general classification of small protrusions with discernable heads (spines) which were likely to be involved in synaptic processes and small protrusions without heads (other protrusions) which were more likely to be growth-associated. This classification does not account for the many structural variabilities and the plasticity of individual protrusions. Finally, differences in network activity, connectivity, and maturation between the OTC model and the intact brain may affect the timing and spatial organization of spinogenesis in GCs. It is plausible that the overall spine density is generally lower and the progression of spinogenesis is slightly delayed in cultured neurons compared to the *in vivo* situation (see Jungenitz et al., 2018). Nevertheless, our observations that early spinogenesis (i.e., ≤ 21 dpi) is evenly distributed along the length of the developing dendritic tree is in line with previous findings in abGCs (Cole et al., 2020; Jungenitz et al., 2018) and consistent with prior work showing that spine formation can arise in the absence of afferent input, as discussed above. Future experiments in the OTC addressing the morphological change of single protrusions over time in combination with enhancement or blockade of synaptic activity might reveal further details about the role of SDPs during the development of pbGCs.

## Data Availability

The raw data supporting the conclusions of this article will be made available by the authors, without undue reservation.

## Footnotes

1
www.treestoolbox.org

2
https://doi.org/10.5281/zenodo.21992440

3
https://inkscape.org
