# Supplementary material for: Early spinogenesis in postnatally-born hippocampal granule cells
Source: Front Neuroanat. 2026 Sep 30;20:1899044. doi: 10.3389/fnana.2026.1899044 (PMC13661989; doi:10.3389/fnana.2026.1899044)
Supplement: Supplementary file 1 [file Supplementary_file_1.docx]

**Figure S1: Anterograde tracing reveals the presence of local and entorhinal cortex (EC) projections in the dentate gyrus (DG) at DIV 10.** Z-projections of confocal image stacks depicting the DG after viral injections of an AAV-hSyn-ChR2-tdTomato into the DG **(A)** or the entorhinal cortex **(B)** at DIV 0 reveal that both the local DG network as well as axonal projections from the EC terminating in the middle and outer molecular layer (MML, OML) are present at DIV 10. The green arrow in **(A)** marks a granule cell extending its mossy fibers to the hilus and dendritic tree into the molecular layer. The red arrow indicates a mossy cell projecting towards the inner molecular layer (IML). Scale bar in **(B)** 150 µm. CA3, cornu ammonis region 3; GCL, granule cell layer.

**Figure S2: Small dendritic protrusions (SDPs) can be observed throughout all parts of the dendritic tree at 12 dpi. (A)** Schematic drawings of four different postnatally-born granule cells at 12 dpi highlighting spines (SDPs with head, indicated in red) and other protrusions (SDPs without head, indicated in blue). Representative images of spines **(B)** and other protrusions **(C)** at 12 dpi. Scale bars in **(A)** 50 µm and **(C)** 0.5 µm.

**Figure S3: Additional data analysis of small dendritic protrusions (SDPs).** Sholl analysis of spines **(A)**, other protrusions **(B)**, and big spines (head area larger than 0.05 µm²) **(C)**. Relative Sholl analysis (for details see methods) of spine tip sizes **(D)** and the density of big spines **(E)**. Densities of spines **(F)**, other protrusions **(G)** and big spines **(H)** were obtained from proximal (p), middle (m), and distal (d) parts of the dendritic tree. **(I)** Relation between the density of big spines in proximal and distal parts of individual pbGCs at 12-20 dpi. Note that densities are normalized to the mean density of each cell. **(K)** Layer-specific analysis of big spines. **(L)** Relation between the density of big spines in the granule cell layer (GCL) and the molecular layer (ML) of individual pbGCs at 12-20 dpi. Number of analyzed cells: 12 and 16 dpi: n = 4; 14,18, 20, and 35 dpi: n = 3. Data are presented as mean values with whiskers indicating SEM. #, number; IML, inner molecular layer; MML, middle molecular layer; OML, outer molecular layer.

**Figure S4:** Methodological process for SDP-Sholl intersection analysis used in Figure 4 and S3.

**Table S5: Detailed statistical reporting for Figure 2I, total dendritic length (pooled).**

| Kruskal-Wallis test |  |  |  |  |  |  |
| --- | --- | --- | --- | --- | --- | --- |
| P value | 0.0031 |  |  |  |  |  |
| Number of groups | 3 |  |  |  |  |  |
| Kruskal-Wallis statistic | 8.959 |  |  |  |  |  |
|  |  |  |  |  |  |  |
| Data summary |  |  |  |  |  |  |
| Number of treatments (columns) | 3 |  |  |  |  |  |
| Number of values (total) | 20 |  |  |  |  |  |
|  |  |  |  |  |  |  |
| Dunn's multiple comparisons test | **Mean rank diff,** | **Adjusted P Value** | **Cohen's d** |  |  |  |
| 12 dpi vs. 14-20 dpi | -8.692 | 0.0305 | 2.94 |  |  |  |
| 12 dpi vs. 35 dpi | -12.33 | 0.019 | 6.53 |  |  |  |
| 14-20 dpi vs. 35 dpi | -3.641 | >0,9999 | 0.79 |  |  |  |
|  |  |  |  |  |  |  |
| Test details | **Mean rank 1** | **Mean rank 2** | **Mean rank diff,** | **n1** | **n2** | **Z** |
| 12 dpi vs. 14-20 dpi | 3 | 11.69 | -8.692 | 4 | 13 | 2.57 |
| 12 dpi vs. 35 dpi | 3 | 15.33 | -12.33 | 4 | 3 | 2.73 |
| 14-20 dpi vs. 35 dpi | 11.69 | 15.33 | -3.641 | 13 | 3 | 0.9609 |

**Table S6: Detailed statistical reporting for Figure 2K, branch points (pooled).**

| Kruskal-Wallis test |  |  |  |  |  |  |
| --- | --- | --- | --- | --- | --- | --- |
| P value | <0,0001 |  |  |  |  |  |
| Number of groups | 3 |  |  |  |  |  |
| Kruskal-Wallis statistic | 12.52 |  |  |  |  |  |
|  |  |  |  |  |  |  |
| Data summary |  |  |  |  |  |  |
| Number of treatments (columns) | 3 |  |  |  |  |  |
| Number of values (total) | 20 |  |  |  |  |  |
|  |  |  |  |  |  |  |
| Dunn's multiple comparisons test | **Mean rank diff,** | **Adjusted P Value** | **Cohen's d** |  |  |  |
| 12 dpi vs. 14-20 dpi | -10.76 | 0.0043 | 2.66 |  |  |  |
| 12 dpi vs. 35 dpi | -2.542 | >0,9999 | 1.25 |  |  |  |
| 14-20 dpi vs. 35 dpi | 8.218 | 0.0894 | 2.04 |  |  |  |
|  |  |  |  |  |  |  |
| Test details | **Mean rank 1** | **Mean rank 2** | **Mean rank diff,** | **n1** | **n2** | **Z** |
| 12 dpi vs. 14-20 dpi | 3.125 | 13.88 | -10.76 | 4 | 13 | 3.187 |
| 12 dpi vs. 35 dpi | 3.125 | 5.667 | -2.542 | 4 | 3 | 0.5636 |
| 14-20 dpi vs. 35 dpi | 13.88 | 5.667 | 8.218 | 13 | 3 | 2.173 |

**Table S7: Detailed statistical reporting for Figure 2L, spatial density (pooled).**

| Kruskal-Wallis test |  |  |  |  |  |  |
| --- | --- | --- | --- | --- | --- | --- |
| P value | 0.003 |  |  |  |  |  |
| Number of groups | 3 |  |  |  |  |  |
| Kruskal-Wallis statistic | 9.045 |  |  |  |  |  |
|  |  |  |  |  |  |  |
| Data summary |  |  |  |  |  |  |
| Number of treatments (columns) | 3 |  |  |  |  |  |
| Number of values (total) | 20 |  |  |  |  |  |
|  |  |  |  |  |  |  |
| Dunn's multiple comparisons test | **Mean rank diff,** | **Adjusted P Value** | **Cohen's d** |  |  |  |
| 12 dpi vs. 14-20 dpi | 5.712 | 0.2736 | 1.24 |  |  |  |
| 12 dpi vs. 35 dpi | 13.58 | 0.0079 | 3.13 |  |  |  |
| 14-20 dpi vs. 35 dpi | 7.872 | 0.1131 | 2.23 |  |  |  |
|  |  |  |  |  |  |  |
| Test details | **Mean rank 1** | **Mean rank 2** | **Mean rank diff,** | **n1** | **n2** | **Z** |
| 12 dpi vs. 14-20 dpi | 16.25 | 10.54 | 5.712 | 4 | 13 | 1.689 |
| 12 dpi vs. 35 dpi | 16.25 | 2.667 | 13.58 | 4 | 3 | 3.007 |
| 14-20 dpi vs. 35 dpi | 10.54 | 2.667 | 7.872 | 13 | 3 | 2.078 |

**Table S8: Detailed statistical reporting for Figure 3B, protrusion density (pooled).**

| Kruskal-Wallis test |  |  |  |  |  |  |
| --- | --- | --- | --- | --- | --- | --- |
| P value | 0.0016 |  |  |  |  |  |
| Number of groups | 3 |  |  |  |  |  |
| Kruskal-Wallis statistic | 9.677 |  |  |  |  |  |
|  |  |  |  |  |  |  |
| Data summary |  |  |  |  |  |  |
| Number of treatments (columns) | 3 |  |  |  |  |  |
| Number of values (total) | 20 |  |  |  |  |  |
|  |  |  |  |  |  |  |
| Dunn's multiple comparisons test | **Mean rank diff,** | **Adjusted P Value** | **Cohen's d** |  |  |  |
| 12 dpi vs. 14-20 dpi | -5.231 | 0.3661 | 1.06 |  |  |  |
| 12 dpi vs. 35 dpi | -14 | 0.0058 | 4.40 |  |  |  |
| 14-20 dpi vs. 35 dpi | -8.769 | 0.062 | 3.91 |  |  |  |
|  |  |  |  |  |  |  |
| Test details | **Mean rank 1** | **Mean rank 2** | **Mean rank diff,** | **n1** | **n2** | **Z** |
| 12 dpi vs. 14-20 dpi | 5 | 10.23 | -5.231 | 4 | 13 | 1.546 |
| 12 dpi vs. 35 dpi | 5 | 19 | -14 | 4 | 3 | 3.098 |
| 14-20 dpi vs. 35 dpi | 10.23 | 19 | -8.769 | 13 | 3 | 2.314 |

**Table S9: Detailed statistical reporting for Figure 3E, tip sizes of spines (pooled).**

| Kruskal-Wallis test |  |  |  |  |  |  |
| --- | --- | --- | --- | --- | --- | --- |
| P value | 0.0006 |  |  |  |  |  |
| Number of groups | 3 |  |  |  |  |  |
| Kruskal-Wallis statistic | 10.49 |  |  |  |  |  |
|  |  |  |  |  |  |  |
| Data summary |  |  |  |  |  |  |
| Number of treatments (columns) | 3 |  |  |  |  |  |
| Number of values (total) | 20 |  |  |  |  |  |
|  |  |  |  |  |  |  |
| Dunn's multiple comparisons test | **Mean rank diff,** | **Adjusted P Value** | **Cohen's d** |  |  |  |
| 12 dpi vs. 14-20 dpi | -7.423 | 0.0846 | 1.68 |  |  |  |
| 12 dpi vs. 35 dpi | -14.5 | 0.004 | 3.84 |  |  |  |
| 14-20 dpi vs. 35 dpi | -7.077 | 0.1855 | 1.95 |  |  |  |
|  |  |  |  |  |  |  |
| Test details | **Mean rank 1** | **Mean rank 2** | **Mean rank diff,** | **n1** | **n2** | **Z** |
| 12 dpi vs. 14-20 dpi | 3.5 | 10.92 | -7.423 | 4 | 13 | 2.194 |
| 12 dpi vs. 35 dpi | 3.5 | 18 | -14.5 | 4 | 3 | 3.209 |
| 14-20 dpi vs. 35 dpi | 10.92 | 18 | -7.077 | 13 | 3 | 1.868 |

**Table S10: Detailed statistical reporting for Figure 3H, tip sizes of other protrusions (pooled).**

| Kruskal-Wallis test |  |  |  |  |  |  |
| --- | --- | --- | --- | --- | --- | --- |
| P value | 0.0153 |  |  |  |  |  |
| Number of groups | 3 |  |  |  |  |  |
| Kruskal-Wallis statistic | 7.216 |  |  |  |  |  |
|  |  |  |  |  |  |  |
| Data summary |  |  |  |  |  |  |
| Number of treatments (columns) | 3 |  |  |  |  |  |
| Number of values (total) | 20 |  |  |  |  |  |
|  |  |  |  |  |  |  |
| Dunn's multiple comparisons test | **Mean rank diff,** | **Adjusted P Value** | **Cohen's d** |  |  |  |
| 12 dpi vs. 14-20 dpi | -6.904 | 0.1238 | 1.39 |  |  |  |
| 12 dpi vs. 35 dpi | -11.75 | 0.0279 | 4.52 |  |  |  |
| 14-20 dpi vs. 35 dpi | -4.846 | 0.6028 | 0.93 |  |  |  |
|  |  |  |  |  |  |  |
| Test details | **Mean rank 1** | **Mean rank 2** | **Mean rank diff,** | **n1** | **n2** | **Z** |
| 12 dpi vs. 14-20 dpi | 4.25 | 11.15 | -6.904 | 4 | 13 | 2.041 |
| 12 dpi vs. 35 dpi | 4.25 | 16 | -11.75 | 4 | 3 | 2.6 |
| 14-20 dpi vs. 35 dpi | 11.15 | 16 | -4.846 | 13 | 3 | 1.279 |

**Table S11: Detailed statistical reporting for Figure 3K, density of big spines (pooled).**

| Kruskal-Wallis test |  |  |  |  |  |  |
| --- | --- | --- | --- | --- | --- | --- |
| P value | <0,0001 |  |  |  |  |  |
| Number of groups | 3 |  |  |  |  |  |
| Kruskal-Wallis statistic | 13.6 |  |  |  |  |  |
|  |  |  |  |  |  |  |
| Data summary |  |  |  |  |  |  |
| Number of treatments (columns) | 3 |  |  |  |  |  |
| Number of values (total) | 20 |  |  |  |  |  |
|  |  |  |  |  |  |  |
| Dunn's multiple comparisons test | **Mean rank diff,** | **Adjusted P Value** | **Cohen's d** |  |  |  |
| 12 dpi vs. 14-20 dpi | -8.5 | 0.0359 | 1.29 |  |  |  |
| 12 dpi vs. 35 dpi | -16.5 | 0.0008 | 2.81 |  |  |  |
| 14-20 dpi vs. 35 dpi | -8 | 0.1043 | 2.47 |  |  |  |
|  |  |  |  |  |  |  |
| Test details | **Mean rank 1** | **Mean rank 2** | **Mean rank diff,** | **n1** | **n2** | **Z** |
| 12 dpi vs. 14-20 dpi | 2.5 | 11 | -8.5 | 4 | 13 | 2.513 |
| 12 dpi vs. 35 dpi | 2.5 | 19 | -16.5 | 4 | 3 | 3.652 |
| 14-20 dpi vs. 35 dpi | 11 | 19 | -8 | 13 | 3 | 2.111 |

**Table S12: Detailed statistical reporting for Figure 3M, percentage of big spines (pooled).**

| Kruskal-Wallis test |  |  |  |  |  |  |
| --- | --- | --- | --- | --- | --- | --- |
| P value | 0.0019 |  |  |  |  |  |
| Number of groups | 3 |  |  |  |  |  |
| Kruskal-Wallis statistic | 9.422 |  |  |  |  |  |
|  |  |  |  |  |  |  |
| Data summary |  |  |  |  |  |  |
| Number of treatments (columns) | 3 |  |  |  |  |  |
| Number of values (total) | 20 |  |  |  |  |  |
|  |  |  |  |  |  |  |
| Dunn's multiple comparisons test | **Mean rank diff,** | **Adjusted P Value** | **Cohen's d** |  |  |  |
| 12 dpi vs. 14-20 dpi | -8.538 | 0.0348 | 1.73 |  |  |  |
| 12 dpi vs. 35 dpi | -13 | 0.012 | 3.12 |  |  |  |
| 14-20 dpi vs. 35 dpi | -4.462 | 0.7171 | 1.03 |  |  |  |
|  |  |  |  |  |  |  |
| Test details | **Mean rank 1** | **Mean rank 2** | **Mean rank diff,** | **n1** | **n2** | **Z** |
| 12 dpi vs. 14-20 dpi | 3 | 11.54 | -8.538 | 4 | 13 | 2.524 |
| 12 dpi vs. 35 dpi | 3 | 16 | -13 | 4 | 3 | 2.877 |
| 14-20 dpi vs. 35 dpi | 11.54 | 16 | -4.462 | 13 | 3 | 1.177 |
